# Supplementary material for: General mechanisms of task engagement in the primate frontal cortex
Source: Nat Commun. 2024 Jun 5;15:4802. doi: 10.1038/s41467-024-49128-w (PMC11153620; doi:10.1038/s41467-024-49128-w)
Supplement: Supplementary file 3 — Reporting Summary [file 41467_2024_49128_MOESM3_ESM.pdf]

nature portfolio

Corresponding author(s): Jan Grohn

Last updated by author(s): Apr 12, 2024

Reporting Summary

Nature Portfolio wishes to improve the reproducibility of the work that we publish. This form provides structure for consistency and transparency in reporting. For further information on Nature Portfolio policies, see our Editorial Policies and the Editorial Policy Checklist.

Statistics

For all statistical analyses, confirm that the following items are present in the figure legend, table legend, main text, or Methods section.

n/a	Confirmed	
☐	☒	The exact sample size (*n*) for each experimental group/condition, given as a discrete number and unit of measurement
☐	☒	A statement on whether measurements were taken from distinct samples or whether the same sample was measured repeatedly
☐	☒	The statistical test(s) used AND whether they are one- or two-sided<br>*Only common tests should be described solely by name; describe more complex techniques in the Methods section.*
☐	☒	A description of all covariates tested
☐	☒	A description of any assumptions or corrections, such as tests of normality and adjustment for multiple comparisons
☐	☒	A full description of the statistical parameters including central tendency (e.g. means) or other basic estimates (e.g. regression coefficient) AND variation (e.g. standard deviation) or associated estimates of uncertainty (e.g. confidence intervals)
☐	☒	For null hypothesis testing, the test statistic (e.g. *F*, *t*, *r*) with confidence intervals, effect sizes, degrees of freedom and *P* value noted<br>*Give P values as exact values whenever suitable.*
☒	☐	For Bayesian analysis, information on the choice of priors and Markov chain Monte Carlo settings
☒	☐	For hierarchical and complex designs, identification of the appropriate level for tests and full reporting of outcomes
☒	☐	Estimates of effect sizes (e.g. Cohen's *d*, Pearson's *r*), indicating how they were calculated

Our web collection on statistics for biologists contains articles on many of the points above.

Software and code

Policy information about availability of computer code

Data collection	No new data was collected for this study. Details of the software used to collect each dataset can be found in the corresponding original publication.
Data analysis	Data was analyzed in MATLAB (R2020a). Neural data were prepared for analysis following a dedicated nonhuman primate fMRI processing pipeline using tools from FSL (6.0), ANTs (2.1.0) and MrCat (https://github.com/neuroecology/MrCat). fMRI data were analysed using FSL's FEAT (6.0).

For manuscripts utilizing custom algorithms or software that are central to the research but not yet described in published literature, software must be made available to editors and reviewers. We strongly encourage code deposition in a community repository (e.g. GitHub). See the Nature Portfolio guidelines for submitting code & software for further information.

Data

Policy information about availability of data

All manuscripts must include a data availability statement. This statement should provide the following information, where applicable:

- Accession codes, unique identifiers, or web links for publicly available datasets
- A description of any restrictions on data availability
- For clinical datasets or third party data, please ensure that the statement adheres to our policy

All datasets used in this study have already previously been published. Please contact the corresponding authors of the original publications for access to the raw datasets. The processed data are available at https://doi.org/10.5281/zenodo.10960864. Source data are provided with this paper.

Research involving human participants, their data, or biological material

Policy information about studies with human participants or human data. See also policy information about sex, gender (identity/presentation), and sexual orientation and race, ethnicity and racism.

Reporting on sex and gender	no human research participants were involved in this study
Reporting on race, ethnicity, or other socially relevant groupings	no human research participants were involved in this study
Population characteristics	no human research participants were involved in this study
Recruitment	no human research participants were involved in this study
Ethics oversight	no human research participants were involved in this study

Note that full information on the approval of the study protocol must also be provided in the manuscript.

Field-specific reporting

Please select the one below that is the best fit for your research. If you are not sure, read the appropriate sections before making your selection.

☒ Life sciences ☐ Behavioural & social sciences ☐ Ecological, evolutionary & environmental sciences

For a reference copy of the document with all sections, see nature.com/documents/nr-reporting-summary-flat.pdf

Life sciences study design

All studies must disclose on these points even when the disclosure is negative.

Sample size	Due to practical and ethical limitations associated with the use of non-human primates in research, we relied on preexisting datasets for this study. For our neuroimaging analyses, we identified data from 13 unique animals across four tasks for a total of 17 datasets that fitted our requirements. This far exceeds the sample sizes commonly employed in the field (2-4 animals). For our TUS analyses, we re-used datasets from four animals, which matches the sample sizes commonly empoyed in the field.
Data exclusions	As we re-used preexisting datasets, all the exclusion criteria of the original studies applied. Additionally, for our analyses of task disengagement, we excluded datasets in which animals disengaged less than a pre-established cut-off of 5% of trials on average across sessions (6 datasets). For our analyses of vigor, we excluded data from one study as it was not possible to compute our measure of vigor for these datasets (4 datasets)
Replication	Our analyses relied on replicating our results across 4 separate study designs / tasks. Each task was performed by multiple animals. Details of the number of animals per task and the number of sessions per animals are given in Supplementary Figure S1.
Randomization	Our study does not include any group specific effects, task or group comparisons. As such, we did not need to apply any randomization. As the data was pre-excisting we could also not assign any specific animal to any particular dataset. The four animals participating in the TUS experiment were all assigned to each stimulation condition.
Blinding	As we did not collect any new data for the study, and our hypotheses were formed post data collection, there was no need for any data acquisition blinding. Furthermore, as we did not do any group comparisons or subject selection based on subjective criteria there was also no blinding we could have done based on group labels during analyses.

Reporting for specific materials, systems and methods

We require information from authors about some types of materials, experimental systems and methods used in many studies. Here, indicate whether each material, system or method listed is relevant to your study. If you are not sure if a list item applies to your research, read the appropriate section before selecting a response.

Materials & experimental systems

n/a	Involved in the study
☒	☐ Antibodies
☒	☐ Eukaryotic cell lines
☒	☐ Palaeontology and archaeology
☐	☒ Animals and other organisms
☒	☐ Clinical data
☒	☐ Dual use research of concern
☒	☐ Plants

Methods

n/a	Involved in the study
☒	☐ ChIP-seq
☒	☐ Flow cytometry
☐	☒ MRI-based neuroimaging

Animals and other research organisms

Policy information about studies involving animals; ARRIVE guidelines recommended for reporting animal research, and Sex and Gender in Research

Laboratory animals	13 rhesus macaques participated in this study. Animals were bred in a UK facility, were aged between 4 and 8 years, and weighted between 8.6 and 14.2 kg.
Wild animals	No wild animals were used
Reporting on sex	As is common practice in the field, mostly male macaques participated in the experiment (12 males, 1 female). As such, no sex based analyses were possible.
Field-collected samples	No field collected samples were used.
Ethics oversight	All procedures were conducted under licenses from the United Kingdom (UK) Home Office in accordance with the UK Animals (Scientific Procedures) Act 1986 and with the European Union guidelines (EU Directive 2010/63/EU).

Note that full information on the approval of the study protocol must also be provided in the manuscript.

Plants

Seed stocks	n/a
Novel plant genotypes	n/a
Authentication	n/a

Magnetic resonance imaging

Experimental design

Design type	Event-related designs
Design specifications	Details on the studies can be found in the original publications. We provide brief summaries here:<br><br>For study 1 (Jahn et al.), each subject performed 37-41 sessions (ranging from 144-561 trials; mean=389.7; sd=79.7). Trials consisted of a wait period (1-2 s), subjects' responses (median RT = 831 ms), a feedback period (3-4 s on first choices, 1-2 s otherwise), an outcome period of 1s, a reward period of 0.4 x reward amount ms, and an inter-choice-intervals of 2-3 s for first choices and 1-2 s otherwise. Additionally, there was a delay of 3-5 s before a new horizon started.<br><br>For study 2 (Grohn et al.) each subject performed 11-13 sessions. Each session consisted of 150 rewarded (i.e. correct trials). Incorrect trials were repeated until the animal made the correct response. Each trial began with a blank screen (2-4s), followed by stimuli presentation and the subjects' responses (median RT = 718 ms), and ended with reward delivery (1.5 s on average).

For study 3 (Bongioanni et al.), each subject performed 12 sessions consisting of 180 trials each. No response trials were included again at the end of a session. For each trial, stimuli were presented up to 60 s, or until a response was made (median RT 952 ms). The inter-trial-interval had a duration of 5-7 s, the action-outcome delay lasted 3.5-4.5 s, and the outcome cue lasted 3 s.

For study 4 (Khaliginejad et al.), each subject performed 22-24 sessions. Sessions finished after 40 m, regardless of the number of trials completed (median RT = 2.28 s). The inter-trial-interval had a duration of 3-7 s, the action-outcome delay was 4s, the outcome was shown for 2 s.

Behavioral performance measures

Button presses and response times were recorded for all studies. For the purpose of this study, we only included animals that failed to respond (i.e. press the button within a response interval) on more than 5% of trials on avearge over sessions within a dataset.

Acquisition

Imaging type(s)

Functional, structural

Field strength

3 Tesla

Sequence & imaging parameters

For each monkey, structural images were acquired under general anesthesia, using a T1-weighted MP-RAGE sequence with a resolution of 0.5 × 0.5 × 0.5 mm, repetition time (TR) = 2.05 s, echo time (TE) = 4.04 ms, inversion time (TI) = 1.1 s, and flip angle of 8°.

fMRI data were acquired using a gradient-echo T2* echo planar imaging (EPI) sequence with the following parameters: 1.5 x 1.5 x 1.5 mm resolution, 36 axial interleaved slices with no gap, TR of 2280 ms, TE of 30 ms and 130 volumes per run. Proton-density-weighted images using a gradient-refocused echo (GRE) sequence (TR = 10 ms, TE = 2.52 ms) were acquired as reference for offline image reconstruction.

Area of acquisition

Whole brain

Diffusion MRI ☐ Used ☐ Not used

Preprocessing

Preprocessing software

Data were preprocessed for analysis following a dedicated nonhuman primate fMRI processing pipeline using tools from FSL (6.0), ANTS (2.1.0), and MrCat (https://github.com/neuroecology/MrCat).

Normalization

The slice-registered average functional image was non-linearly registered to the high-resolution structural reference of each subject, and then this was registered to the template using tools in ANTS as implemented in MrCat.

Normalization template

We used a template in F99 space (Van Essen 2002).

Noise and artifact removal

To correct for non-linear motion-related artefacts in the phase-encoding direction due to body movement, each slice was registered, first linearly and then non-linearly, to a robust reference based on EPI volumes from the same timeseries with least distortion, using a processing pipeline implemented in MrCat. The functional images were temporally filtered with high-pass filters (cutoff of 100s), and spatially smoothed with Gaussian spatial smoothing (FWHM of 3mm)

Volume censoring

During analyses, EPI volumes suffering from strong artefacts were excluded. Volume quality was accessed based on slice registration cost, linear scaling along the phase-encoding direction, and non-linear deformation.

Statistical modeling & inference

Model type and settings

We used a univariate approach within the GLM framework as implemented in FSL's FEAT. Overall, we had a three level hierarchy with the first being all datapoints in an individual session, second all sessions of an individual animal within a task and the third across tasks. We used Fixed effects models for all within subject analyses (first to second) and random effects (FLAME 1+2) for the between subject analyses on the third level.

Effect(s) tested

We included regressors for the residuals after regressing out the task, and the filtered residuals. The filtered residuals were included once with a filter that weighted the history of residuals, and once with a filter that weighted the upcoming residuals. We did this both for residuals obtained by predicting disengagements, and by predicting response times.

We used contrasts to examine the overall filtered residuals (past+future), the sum of the residuals with the filtered residuals (past+future), the difference between the residuals and the filtered residuals (past+future), and the difference between past-future. We did this both for residuals obtained by predicting disengagements, and by predicting response times.

Specify type of analysis: ☐ Whole brain ☐ ROI-based ☒ Both

Anatomical location(s)

Anatomical regions were defined according to an atlas by Reverley et al., 2017. Our ROIs are defined as the overlap between the anatomical region and functional activation.

Statistic type for inference

(See Eklund et al. 2016)

Clusters were determined using a threshold of z>2.3

Correction	FWE-correction threshold of p = 0.05

Models & analysis

n/a	Involved in the study
☒	☐ Functional and/or effective connectivity
☒	☐ Graph analysis
☒	☐ Multivariate modeling or predictive analysis
